# Leitfaden Kurzdarmsyndrom

**DOI:** 10.1055/a-2375-4601

**Published:** 2025-05-13

**Authors:** Stefanie Dabsch, Christian Datz, Clemens Dejaco, Felix Harpain, Elisabeth Hütterer, Ludwig Kramer, Nina Loschko, Alexander Moschen, Anton Stift, Harald Vogelsang

**Affiliations:** 1 27271 Klinische Abteilung für Gastroenterologie und Hepatologie, Universitätsklinik für Innere Medizin 3, Medizinische Universität Wien, Vienna, Austria; 2 31507 Abteilung für Innere Medizin, Krankenhaus Oberndorf, Lehrkrankenhaus der Paracelsus Medizinischen Privatuniversität Salzburg, Salzburg, Austria; 3 27271 Klinische Abteilung für Viszeralchirurgie, Universitätsklinik für Allgemeinchirurgie, Medizinische Universität Wien, Vienna, Austria; 4 27271 Klinische Abteilung für Onkologie, Universitätsklinik für Innere Medizin 1, Medizinische Universität Wien, Vienna, Austria; 5 603639 1. Medizinische Abteilung, Wiener Gesundheitsverbund Klinik Hietzing, Wien, Austria; 6 27266 Universitätsklinik für Innere Medizin 2, Johannes Kepler Universität Linz, Linz, Austria

**Keywords:** Chronisches Darmversagen, Teduglutid, Heim-parenterale Ernährung, Kurzdarmsyndrom, Chronische Darminsuffizienz, intestinal failure, teduglutide, home parenteral nutrition, short bowel syndrome, intestinal insufficiency

## Abstract

Das Kurzdarmsyndrom ist ein seltenes und komplexes Krankheitsbild, das meist aufgrund von ausgeprägter Dünndarmresektionen entsteht und zu einem chronischen Darmversagen führen kann. Durch die verminderte intestinale Resorptionsoberfläche kommt es zu einer reduzierten Aufnahme von Makro-, Mikronährstoffen und/oder Flüssigkeit. Dementsprechend entstehen vielfältige Symptome wie Durchfall, Gewichtsverlust, Vitaminmangelerscheinungen, Niereninsuffizienz, Hepatopathie und andere mit großem Effekt auf die Lebensqualität dieser Patienten. Die Therapie dieser Patienten ist komplex und bedarf einer interdisziplinären Zusammenarbeit von verschiedenen Fachdisziplinen wie Diätologie, Gastroenterologie, Chirurgie sowie die zusätzliche engmaschige Betreuung im niedergelassenen Bereich. Oft brauchen Patienten eine dauerhafte (heim-)parenterale Unterstützung. Therapien müssen individuell entschieden und regelmäßig auf Effektivität und Nebenwirkungen überprüft werden. Weiter sind regelmäßige Bestimmungen verschiedener klinischer und laborchemischer Parameter notwendig. Die Morbidität und Mortalität dieser Erkrankung ist hoch und vom Auftreten von Komplikationen geprägt. Dieser Leitfaden soll einen Überblick über die Erkrankung sowie die notwendige Diagnostik und Therapieoptionen geben und die bestmögliche Betreuung dieser komplexen Patienten ermöglichen.

## Abstract (en)

Short bowel syndrome is a rare complex disease that mainly develops due to extensive bowel resections and can lead to chronic intestinal failure. Due to the decreased intestinal surface absorption of macronutrient, micronutrient and/or fluids is reduced. Correspondingly manifold symptoms arise as diarrhoea, weight loss, vitamin deficiencies, chronic kidney disease, hepatopathy and others with great impact on quality of life of patients. Therapy is complex and needs interdisciplinary collaboration between dieticians, gastroenterologists, surgeons and also a dense monitoring of general practitioners. Commonly patients need permanent home-parenteral support. Therapies have to be decided individually and have to be reviewed regularly for effectivity and side effects. Furthermore, periodic monitoring of several clinical and laboratory tests should be performed. Morbidity and mortality of this disease complex is high and lead by appearance and management of complications. This practical guide should give an overview about the disease, diagnostics and management and should enable the best possible care of these complex patients.

## Einleitung

Das Kurzdarmsyndrom (KDS) ist ein seltenes Krankheitsbild, bei dem es zu einer chronisch reduzierten Funktion des Dünndarms kommt. Es ist definiert durch eine verbleibende Dünndarmlänge in continuitatem von weniger als 200 cm und ist meist hervorgerufen durch extensive Resektionen 1 . Man unterscheidet zwischen intestinaler Insuffizienz, wenn Patienten mit optimaler pharmakologischer und diätologischer Therapie die Verluste kompensieren können, und intestinalem Versagen, wenn Patienten nur mit parenteraler Unterstützung überleben können. Ein Versagen des Dünndarms führt nicht nur zu vielfältigen Symptomen mit massiv reduzierter Lebensqualität, sondern hat auch schwere Auswirkungen auf andere Organe wie Nieren oder Leber 2 .

### Ursachen

Die Ursachen sind sehr heterogen ( Tab. 1 ). Es gibt sehr selten angeborene Ursachen wie die Dünndarmatresie und Malrotationen, welche zu einem Volvulus mit nachfolgender Ischämie führen kann. Meist sind die Ursachen erworben und führen zu ausgeprägten Darmresektionen. Ein Drittel der Patienten umfasst Kinder, nur 20% der KDS entstehen außerhalb der Neugeborenenpopulation. Neben den oben erwähnten angeborenen Erkrankungen ist die nekrotisierende Enterokolitis in ca 30% schuld am KDS im Kindesalter 3 . Die mesenteriale Ischämie (venös oder arteriell) stellt mit rund einem Drittel eine der häufigsten Ursachen im Erwachsenenalter dar, ein weiteres Drittel umfasst Patienten mit Morbus Crohn, eine chronisch-entzündliche Darmerkrankung mit oft wiederholt notwendigen Darmresektionen 4 . Selten kann die Ausbildung intestinaler Fisteln (entero-colonisch, entero-cutan, entero-enteral) postoperativ oder im Rahmen eines Morbus Crohn und der damit verbundene Verlust der Resorptionsstrecke zu einem funktionellem KDS führen. Jede abdominelle Operation birgt ein potenzielles Risiko für chirurgische Komplikationen wie Blutungen, Anastomosendehiszenzen oder Verletzungen der Darmwand, welche zu Revisionen mit neuerlichen Darmresektionen und schlussendlich zu einem KDS führen können. Diese stellen mit knapp 15% einen relativ großen Anteil an diesem Patientenkollektiv dar 2 5 6 .

**Table:** **Tab. 1** Häufigste Ursachen für ein Kurzdarmsyndrom bei Kindern und Erwachsenen.

Kinder	Erwachsene
Nekrotisierende Enterocolitis	Mesenteriale Ischämie
Malrotation	Morbus Crohn
Intestinal Atresie	Chirurgische Komplikationen
	Strahlenenteritis
	Volvulus

### Prävalenz/Inzidenz

Die weltweite Prävalenz und Inzidenz des KDS ist unbekannt, da es an verlässlichen Daten mangelt. Annähernde Schätzungen können auf Grundlage von Patienten mit Langzeit-parenteraler Ernährung vorgenommen werden. In Europa geht man von einer Prävalenz von 1,4 Fällen pro 1 Million Einwohner aus, steigend in den letzten Jahren 7 , wobei hier deutliche Unterschiede in den einzelnen Ländern zu verzeichnen sind (z.B. KDS-Prävalenz in Polen 0,4/1 Mio. Einwohner vs. 30/1 Mio. Einwohner in Dänemark) 8 9 . Dies liegt vor allem an der unterschiedlichen Dichte von spezialisierten Zentren und der unterschiedlichen Verfügbarkeit von heimparenteraler Ernährung, was in weiterer Folge zu einem „Underreporting“ bzw. einer Unfähigkeit der adäquaten Diagnostik und Behandlung der betroffenen Patienten führt.

### Anatomie

**Abb. 1:**
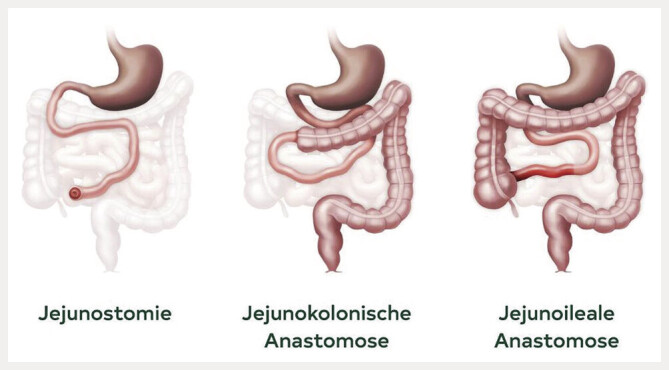
Typen nach Messing, Typ 1 Jejunostomie, Typ 2 Jejunokolonische Anastomose, Typ 3 erhaltene Ileocoekalklappe sowie erhaltenes Colon. Daten nach 11 . Quelle Takeda Pharma Ges.m.b.H., Wien.

Der gesunde Dünndarm hat eine ungefähre Länge von 300–400 cm 10 , das Colon eine Länge von ungefähr 150 cm. Der Schweregrad und somit die Prognose ist stark von den verbleibenden Darmabschnitten abhängig. Man unterscheidet grob drei anatomische Subtypen des KDS: Typ 1, die endständige Jejuno/Ileostomie; Typ 2 die jejuno/ileokolonischen Anastomose und Typ 3 die jejuno-ileale Anastomose mit erhaltener Ileozökalklappe und in Gänze erhaltenem Colon 11 12 13 ( Abb. 1 ). Eine Abhängigkeit von parenteraler Unterstützung ist nahezu 100% bei einer Restdünndarmlänge von <90 cm beim Typ 1, <60 cm beim Typ 2 und <30 cm beim Typ 3 11 14 . Diese Unterschiede resultieren daher, dass die verbliebenen Darmabschnitte sich physiologisch unterschiedlich gut an die veränderte Situation adaptieren können. So kann es vor allem im Ileum zu einem Zotten- und Kryptenwachstum sowie vergrößertem Darmdurchmesser und -länge und auch wahrscheinlich einer zusätzlich funktionellen Verbesserung kommen, was insgesamt zu einer verbesserten Flüssigkeits- und Nährstoffaufnahme führt. Das Colon spielt eine große Rolle in der Resorption von Flüssigkeiten, Elektrolyten und kurzkettigen Fettsäuren. Die Metabolisierung von unverdauten Kohlenhydraten durch das kolonische Mikrobiom zu Fettsäuren und deren Aufnahme stellt eine wichtige Energiequelle für KDS-Patienten dar und kann bis zu 50% des Energietagesbedarfes decken 15 16 .

### Pathophysiologie

Die primäre Konsequenz des KDS ist eine Reduktion der absorptiven Oberfläche des Darms mit nachfolgender Malabsorption abhängig nicht nur von der verbleibenden Anatomie sondern auch von Erkrankungen des verbliebenen Darmes (z.B. Strahlenenteritis, Morbus Crohn) 17 . Die Malabsorption führt zu Einschränkungen der Makro- (Kohlenhydrate, Proteine, Fette) und Mikronährstoffe (Elektrolyte, Vitamine, Spurenelemente) und/oder der Flüssigkeitsaufnahme 1 12 .

Der postoperative Adaptationsprozess wird in drei Phasen eingeteilt 6 13 18 : Die akute Phase (hypersekretorische Phase) beginnt unmittelbar nach der intestinalen Resektion, dauert in der Regel wenige Wochen (bis Monate) und ist vor allem durch massive enterale Verluste von Flüssigkeit, Elektrolyten und Nährstoffen geprägt. Dies entsteht durch gestörte neurohumorale Regulationsmechanismen, Veränderung der Motilität und veränderte Feedback-Mechanismen. In den darauffolgenden ein bis zwei Jahren schließt sich ein physiologischer Adaptionsprozess an, in der das verbleibende Intestinum zunehmend absorptive Funktionen fehlender Darmabschnitte übernimmt (Adaptionsphase). Es kommt unter anderem zu strukturellen Anpassungen der Dünndarmmukosa mit Hypertrophie der Zotten und Vertiefung der Krypten 19 . Wenige Jahren postoperativ ist das Maximum der Adaption erreicht und eine weitere Verbesserung der Darmfunktion selten (stabile Phase): In einer Studie von Jeppesen et al liegt die Wahrscheinlichkeit eines permanenten intestinalen Versagens nach zwei Jahren mit intravenöser Abhängigkeit bei etwa 94% 11 . Andere Untersuchungen zeigten, dass es auch danach, bei einem geringen Anteil von Patienten, zu einer oralen Autonomie kommen kann vor allem bei Patienten mit längerem erhaltenem Colon, einer Dünndarmlänge von >75cm und bei höheren Plasma Citrullin-Konzentration 20 . Schlussendlich kommt es zu einer stabilen Phase in der vor allem akute Exazerbationen und Komplikationen vermieden werden sollen.

### Klinik

Die klinische Präsentation der KDS-Patienten ist aufgrund der anatomischen Unterschiede und verschiedenen Grunderkrankungen sehr heterogen. Symptome können Durchfall, Müdigkeit, Schlafprobleme, Unterernährung/Gewichtsverlust, Dehydrierung, Bauchschmerzen/-krämpfe, Blähungen sowie metabolische Entgleisungen sein 21 . Die Patienten präsentieren sich mit Hunger- und Durstgefühl und Symptomen von Mangelerscheinungen wie Muskelkrämpfen, Konzentrationsschwäche, Nachtblindheit, Müdigkeit je nach fehlendem Mikronährstoff. Besonders häufig ist ein Mangel an fettlöslichen Vitaminen A, D, E und K sowie ein Mangel an Magnesium und Kalium ( 22 sowie expert opinion). Chronische Mangelerscheinungen können auch Langzeitfolgen wie neurologische Probleme, Nachtblindheit oder Hautprobleme verursachen.

## Diagnostik

Die Diagnose erfolgt meist intraoperativ (die Restdünndarmlänge sollte in jedem OP-Bericht enthalten sein) oder postoperativ mittels radiologischer Methoden (Enterografie-MRT/CT) und wird gestellt bei einer verbleibenden Dünndarmlänge (gemessen aboral der Flexura Duodenojejunalis) von <200 cm 23 . Um die Prognose besser abschätzen zu können, ist eine genaue Kenntnis und Dokumentation der verbleibenden Anatomie und Darmlänge essenziell. Studien zeigen, dass die postoperative bildgebende Vermessung hierbei gut mit der intraoperativen Vermessung korreliert, speziell bei Darmlängen <200–250 cm 24 25 26 .

### Laborkontrollen und klinische Kontrollen ( Tab. 2 )

**Table:** **Tab. 2** Empfohlene Kontrollintervalle diverser Parameter bei stabilem Verlauf.

Laborparameter		Klinische Parameter	
**Parameter**	**Häufigkeit**	**Parameter**	**Häufigkeit**
Blutbild, CRP, Elyte, Niere, Leber, Albumin	Alle 3 Monate	Gewicht	Bei jeder Kontrolle
Vitamin D, Parathormon	Alle 3 Monate	Harnmenge/24h	Bei jeder Kontrolle
Harn-Natrium (Spontanharn)	Alle 3 Monate	Stuhlmenge/frequenz	Bei jeder Kontrolle
Venöses Blutgas (pH, Bikarbonat)	Alle 6 Monate	Bioimpedanzmessung	Alle 6–12 Monate
Vitamin B12, Folsäure	Alle 6 Monate	Knochendichte	Alle 2 Jahre
Eisenstatus	Alle 6 Monate	Citrullin	Optional
Zink, Selen	Alle 6–12 Monate	D-Xylose-Test	Optional je nach Phase
Vitamin A, Vitamin E	Alle 6–12 Monate	Abdomen Sonographie	Jährlich bei parenteraler Ernährung
Gerinnung (Vitamin K)	Alle 6–12 Monate		

Eine regelmäßige Kontrolle verschiedener klinischer und laborchemischer Parameter ist empfohlen, um Mangelerscheinungen und Komplikationen frühzeitig zu diagnostizieren und dementsprechend behandeln zu können 23 . Eine klinische Untersuchung des Patienten hinsichtlich Allgemeinzustand, Ausfuhr (Stuhlmenge/24h, Harnmenge/24h), Einfuhr (Trinkmenge, Mahlzeiten, parenterale Einfuhr), Körpergewicht, Flüssigkeitshaushalt (Ödeme, Dehydrierungszeichen) sowie Anzeichen Katheter-assoziierter Komplikationen (Fieber/Schüttelfrost, Kathetereinstichstelle) soll bei jeder Visite durchgeführt werden 23 27 . Ergänzend ist die Durchführung einer Bioelektrischen Impedanzanalyse, so verfügbar, empfohlen 28 . Diverse Laborparameter sollten in regelmäßigen Abständen kontrolliert werden, mit gesteigerter Frequenz bei Dekompensationen, postoperativ, bei Start/Reduktion einer parenteralen Ernährung und bei Therapiestart mit Wachstumshormonen.

### Bildgebung

Die Enterografie-CT oder MRT wird eingesetzt zur postoperativen Messung der Darmlänge sowie bei Verdacht auf Komplikationen wie Stenosen. Auch vor Start eines Wachstumshormons sollte eine Bildgebung des Dünndarmes mittels Abdomen-Sonografie oder Enterografie CT/MRT durchgeführt werden um Strikturen, „blind loops“ und unklare Anatomien abzuklären 23 . Bei zugrunde liegendem M. Crohn sollte ebenso eine regelmäßige Bildgebung zur Evaluierung der Entzündungsaktivität stattfinden (CT/MRT/Sonografie, siehe ECCO Guidelines).

### Zusätzliche Diagnostik

Bei Patienten mit grenzwertiger Darmlänge und chronischen Durchfällen kann eine Bestimmung des Citrullin-Spiegels im Plasma oder ein Xylose-Test durchgeführt werden, um die resorptive Kapazität des Restdarmes zu objektivieren 20 29 30 . Beim Xylose-Test werden 25g Xylose (ein vom Menschen nicht abbaubarer Zucker) verabreicht und die Spiegel im Serum und Harn (gesammelt über 5 Stunden) gemessen. Bei normaler resorptiver Kapazität findet sich ca. 20–30% der verabreichten Menge Xylose im Harn wieder. Problematisch ist derzeit eine sehr reduzierte Verfügbarkeit des Tests in Österreich sowie falsch niedrige Werte bei Vorliegen eines small intestinal overgrowth syndroms oder stark eingeschränkter Nierenfunktion.

## Therapie

Der Therapiebedarf ändert sich im Rahmen der natürlichen Adaptionsprozesse postoperativ und muss somit im Laufe der Krankheitsphasen regelmäßig angepasst werden. Die Therapieziele beinhalten klinische Parameter wie normales Körpergewicht, Harnmenge (>1000ml/24h), die Vermeidung von Hunger- und Durstgefühl sowie die Behandlung und Vermeidung von Mangelzuständen (Makro- und Mikronährstoffe) und Komplikationen.

In der hypersekretorischen Phase steht das Management der meist großen Flüssigkeits- und Elektrolytverluste im Vordergrund. Oft sind erhebliche Mengen an parenteraler Flüssigkeit notwendig (4–8L/d!). Protonenpumpen-Hemmer oder H2-Antagonisten helfen, gastrale Hypersekretion zu reduzieren. Diese sollten im Krankheitsverlauf wieder reduziert und abgesetzt werden (so keine andere Indikation vorhanden), da ein langfristiges Fehlen der Magensäure negative Effekte auf die Verdauung und das Mikrobiom (höheres Risiko eines bakteriellen Überwuchses) haben kann 22 . Bei einem high-output Stoma (>2L/d), insbesondere kurzfristig nach Resektionen soll die orale Flüssigkeitszufuhr und Ernährung auf ein absolutes Minimum reduziert werden. Weiter kann eine Therapie mit Octreotid oder Somatostatin in dieser Phase die Flüssigkeitsverluste dezimieren. Diese Therapie sollte engmaschig kontrolliert und nur kurzfristig gegeben werden da es die langfristige Darmadaption negativ beeinflussen kann 22 .

Andere medikamentöse Therapien beinhalten motilitätshemmende Medikamente wie Loperamid, welches in höheren Dosen als üblich gegeben werden kann (bis zu 32mg/d) 22 23 . Loperamid sollte einer Therapie mit Opiaten wie Tinctura opii oder Codein vorgezogen werden, da es weder sedativ ist noch abhängig macht. Der Effektivität von Racecadotril bei KDS ist derzeit noch nicht untersucht worden, ein Therapieversuch bei Versagen anderer Therapien ist eine Option. Bei erhaltenem Colon kann Colestyramin zur Reduktion der Gallensäurenverlust-assoziierten Durchfälle versucht werden, jedoch kann dies auch zu vermehrter Steatorrhoe führen. Umgekehrt kann eine Gallensäure-Ersatztherapie (mit z.B. Cholyl-Sarkosin) die Fett-Absoprtion verbessern 31 , jedoch ist die Verfügbarkeit in Österreich leider sehr eingeschränkt. Auch eine Kombination oben genannter anti-sekretorischer bzw. motilitätshemmender Medikamente kann individuell gegeben werden. Auf jeden Fall sollte bei all diesen Therapien die klinische Wirksamkeit überprüft werden und, falls nicht wirksam, die Therapie wieder abgesetzt werden. Die Gabe von Pankreasenzymen zu jeder Mahlzeit soll die Fettverdauung optimieren (auch bei normaler Pankreasfunktion) und kann allen Patienten empfohlen werden.

**Abb. 2:**
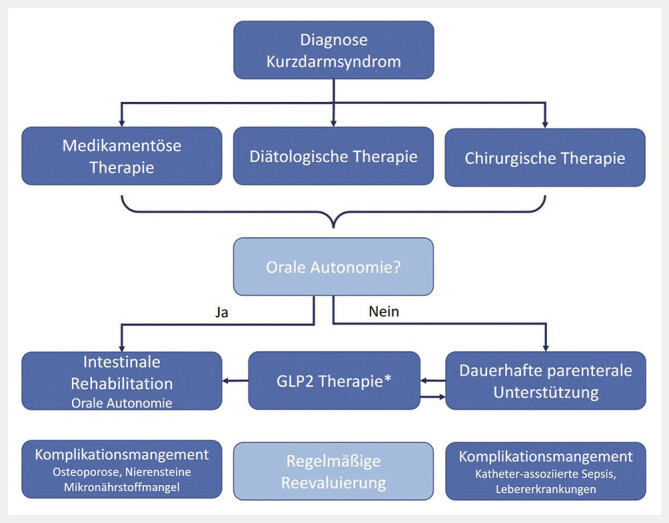
Therapiealgoritmus. *Kontraindikationen: aktive Tumorerkrankung. GLP2 = Glucagon like peptide 2.

Sollte nach Stabilisierung der Adaptionsprozesse und trotz Optimierung der diätologischen und medikamentösen Therapien weiterhin eine Abhängigkeit von parenteraler Unterstützung bestehen, kann eine Therapie mit intestinalen Wachstumsfaktoren in Betracht gezogen werden ( Abb. 2 ) 23 . Der Zeitpunkt des Therapiestarts ist mitunter abhängig von der verbleibenden Anatomie und der dementsprechend zu erwartenden Prognose. Vor Therapiestart sollten Kontraindikationen ausgeschlossen werden. Diese beinhalten eine aktive (oder suszipierte) maligne Erkrankung oder Malignome des Gastrointestinal-Trakts innerhalb der letzten 5 Jahre. Derzeit verfügbar ist das Wachstumshormon Teduglutid, ein kurzwirksames Analogon von Glucagon-like-peptide 2 32 33 . Es führt zu einem Wachstum der Zotten und Krypten und somit zu einer größeren Resorptionsfläche 34 . Weiter wird der enterale Blutfluss erhöht und die Magenentleerung und -säureproduktion reduziert. Nebenwirkungen beinhalten Übelkeit, Bauchschmerzen, Hyperhydratation, Auftreten eines Sub-/Ileus und andere. Nach Durchführung der notwendigen Voruntersuchungen (siehe nachfolgender Absatz) kann der Therapiestart erfolgen 23 . Engmaschige Kontrollen sollten danach durchgeführt werden, um die bestehende parenterale Unterstützung entsprechend zu adaptieren. Da die Effekte nach Absetzen rasch nicht mehr nachweisbar sind, muss eine lebenslange Therapie erfolgen.

### Diagnostik vor Start eines Wachstumshormons

Vor Einleitung einer Therapie mit einem Wachstumshormon soll eine Durchuntersuchung mittels Gastroskopie, Koloskopie (so ein verbliebenes Colon vorhanden – in continuitatem oder „blind“) sowie eine Abdomen-Sonografie inklusive Darmschall oder eine Enterografie CT/MRT erfolgen 23 . Dies dient dem Ausschluss von neoplastischen Erkrankungen und Polypen sowie zur Klärung der Anatomie und Abklärung von Strikturen. Die Gastroskopie, Koloskopie sowie die Abdomen-Sonografie sollten ein Jahr nach Therapiestart sowie in weiterer Folge alle 3–5 Jahre wiederholt werden, solange die Therapie mit dem Wachstumshormon weiterführt wird 23 .

Chirurgische Therapieoptionen sind für ausgewählte Patienten sinnvoll. Falls ein signifikanter Anteil von Dünndarm oder Colon noch erhalten aber nicht „in continuitatem“ ist, sollte eine intestinale Rekonstruktion (mit oder ohne finalem Stoma) angestrebt werden, insbesondere, wenn ein Typ 1 in einen Typ 2 oder 3 übergeführt werden kann. Dies kann in weiterer Folge zu einer enteralen Autonomie führen. Eine Sanierung von sehr hohen Dünndarmfisteln kann ebenso hilfreich sein. Eine Technik, die der Verlängerung des bestehenden Darmes dienen soll, ist die STEP (serial transverse enteroplasty) sowie die LILT extension (longitudinal intestinal lengthening and tailoring nach Bianchi). Beide Methoden werden unseres Wissens nach in Österreich sehr eingeschränkt angeboten. Weiter dient die Chirurgie der Behandlung von Komplikationen wie Adhäsionen, Stenosen etc. Geplante Operationen sollten unbedingt nur an spezialisierten Zentren erfolgen 23 .

Intestinale Transplantationen werden derzeit in Europa sehr selten durchgeführt. Im Jahr 2023 wurden im Eurotransplant-Raum 4 Patienten an 3 Zentren in Deutschland, Belgien und Niederlande transplantiert, davon 3 als Multiviszeraltransplantation. Gründe sind einerseits eine sehr hohe peri- und postoperative Morbidität (und Mortalität), das Problem der technischen Machbarkeit bei multiplen Voroperationen und die chirurgischen technischen Anforderungen. Indiziert wäre die Transplantation in 1. Linie bei Patienten mit fortschreitender intestinal-failure-associated liver disease oder bei fehlendem Venenzugangsmöglichkeiten (Thrombosen der großen Venen). Wenn transplantiert wird, wird meist eine kombinierte Transplantation durchgeführt (Leber, Pankreas und Dünndarm).

## Ernährung

Jeder Patient mit chronischem Darmversagen sollte von einem spezialisierten Diätologen/in mitbetreut werden. Dieser stellt nicht nur eine etwaige parenterale Ernährung ein, sondern optimiert auch die orale Ernährung, welche einen großen Effekt auf den Krankheitsverlauf haben kann. Diese Begleitung sollte lebenslang erfolgen. Ziel der Ernährungstherapie ist ein normaler Ernährungszustand ohne Mangelerscheinungen. Auch ein erhöhter Bedarf wie in Stresssituationen, bei Sportaktivitäten oder Schwangerschaft soll abgedeckt werden können 35 .

Im Vordergrund sollte die orale/enterale Ernährung stehen. Wenn der Bedarf auf diese Art nicht gedeckt werden kann, wird eine parenterale Unterstützung notwendig. Mikronährstoffmängel werden ebenso in erster Linie versucht, oral auszugleichen, nicht immer sind Medikamente verfügbar, teilweise muss auf Nahrungsergänzungsmittel zurückgegriffen werden.

**Table:** **Tab. 3** Substitutionsempfehlungen Vitamin und Spurenelemente (adaptiert von AGA Clinical Practice Update 2022).

Mikronährstoff	Parameter	Typische Supplementation
Vitamin A	Serum Retinol	Oral 5000–50 000 IU täglich, i.m. Administration verfügbar
Vitamin B12	Serum Vitamin B12, Holotranscobalamin	s.c./i.m.: 300–1000 mg monatlich; oral, intranasal und in Schmelztablettenform
Vitamin C	Serum Vitamin C	Oral: 200–500 mg täglich; i.v. auch verfügbar
Vitamin D	Serum 25-Hydroxy Vitamin D, Parathormon, Serum 1–25 Dihydroxy-Vitamin D	Oral: bis 50 000 IU einmal wöchentlich (oder Calcifediol über die internationale Apotheke)
Vitamin E	Serum Vitamin E	Oral: 400 IU bis zu 3×/d
Folsäure	Serum Folsäure, Erythrozyten-Folsäure	Oral 1mg täglich
Eisen	Serum Ferritin, Transferrin-Sättigung, solubler Transferrin-Rezeptor	Oral maximal 100mg täglich oder jeden 2. Tag, Alternativ i.v.
Zink	Serum Zink	Oral 50 mg elementares Zink (220 mg tablet) ein oder zweimal täglich
Selen	Serum Selen	Oral 100–200 mg täglich
Chrom	Serum Chrom	Oral 100–200 mg bis 3× täglich
Kupfer	Serum Kupfer	Oral 2 mg (oder mehr) elementares Kupfer täglich, i.v. verfügbar
IU=internationale Unit; i.m. = intramuskulär; s.c.= subcutan; i.v.=intravenös		

In der Hypersekretionsphase ist in der Regel eine parenterale Gabe von Flüssigkeit, Makro- und Mikronährstoffen notwendig. In dieser Phase sollte die orale Flüssigkeitsmenge limitiert werden, um die Stuhlmenge zu reduzieren. Nach initialer postoperativer Stabilisierung sollte ein früher enteraler Nahrungsaufbau (oral oder mittels Duodenal-/Jejunalsonde) begonnen werden. In weiterer Folge kann die parenterale Unterstützung reduziert und die enterale Aufnahme gesteigert werden. Das Ausschleichen der parenteralen Unterstützung beginnt mit einer verminderten Kalorienmenge bei gleichbleibender Flüssigkeitsgabe und erst in weiterer Folge erfolgt die Reduktion der Flüssigkeitsmenge und der Mikronährstoffe. Auch eine alleinige Gabe von Flüssigkeit und Mikronährstoffen kann langfristig erforderlich sein. Die intravenöse Ernährung sollte möglichst Glucose-reduziert erfolgen, da die Glucose von allen Makronährstoffen enteral am einfachsten resorbiert wird. Ein Blutzucker von 180mg/dL (Nierenschwelle) sollte nicht überschritten werden. Im Gegenteil zur physiologischen oralen Ernährung ist die parenterale Ernährung nicht Bolus-weise, sondern kontinuierlich und enthält ausschließlich Glucose. Diese Faktoren zusammen mit bestehendem Diabetes mellitus oder einer Insulin-Resistenz kann zu einer nicht kontrollierten diabetischen Stoffwechsellage führen trotz maximaler Reduktion der Glucosemenge in den Ernährungsbeuteln und zuckersenkender Medikamente inklusive injiziertem Insulin. In diesem Fall kann dem Ernährungsbeutel kurzwirksames Insulin hinzugefügt werden 23 . Die Menge richtet sich nach dem individuellen Insulin-Bedarf sowie der Menge an Glucose.

Als Lipidemulsionen sollen ausschließlich Mischfette mit Omega-3-Fettsäuren verwendet werden. Die Proteinzufuhr ist so zu wählen, dass Normalwerte im Labor erzielt werden können, ohne die Nierenfunktion negativ zu beeinflussen.

Jedem Ernährungsbeutel sind Vitamine und Spurenelemente in normaler (oder bei Bedarf doppelter Standarddosis) hinzuzufügen. In Ausnahmefällen sind auch deutlich höhere Dosen möglich. Als Standardflüssigkeitslösung eignet sich eine isotone Lösung wie Elomel isoton oder Ringer-Laktat. Die Menge ist abhängig vom individuellen Bedarf und sollte anhand der Harnmenge (zumindest 1000ml/24h) sowie des Durstgefühls gesteuert werden.

Nach jeder Ernährungsinfusion sollte der zentrale Zugang mit physiologischer Kochsalzlösung pulsativ gespült und anschließend mit Taurolidin geblockt werden speziell, wenn schon einmal eine Infektion eines zentralen Katheters vorlag 35 . Bei nächster Verwendung des Katheters soll das Taurolidin nicht aspiriert werden, sondern mittels physiologischer Kochsalzlösung langsam injiziert werden. Bei einer Aspiration käme es zu einer Füllung des Katheters mit Blut, was das Risiko für eine Infektion erhöhen würde. Nur bei Verdacht auf Biofilm-Bildung, Katheterinfektion oder einer (sehr selten auftretenden) Unverträglichkeitsreaktion sollte das Taurolidin aspiriert werden.

Als zentraler Katheter sollte bei zu erwartender Langzeit-Therapie ein voll implantierter oder getunnelter Katheter gewählt werden 35 . Jedoch ist zu beachten, dass bei einem voll implantierten Katheter immer wieder Tage ohne liegende Nadel möglich sein sollten, um lokale Komplikationen zu vermeiden. Mit einem getunnelten Katheter (z.B. Hickmann-Katheter) dürfen Patienten nur dann baden oder schwimmen, wenn durch einen (Spezial-) Verband eine dichte Abdeckung des Katheters gewährleistet werden kann. Falls eine Verbesserung der Darmfunktion erwartet werden kann oder nur eine kurzfristige parenterale Ernährung wahrscheinlich ist (z.B. Bridging bis zur intestinalen Rekonstruktion), kann auch ein peripher eingeführter zentraler Katheter (PICC) verwendet werden. Dieser birgt ein signifikant höheres Risiko einer Venenthrombose und muss nach spätestens 6 (–12) Monaten (bei komplikationslosem Verlauf) gewechselt oder entfernt werden 35 . Ein PICC hat den großen Vorteil einer sehr einfachen Implantation und Explantation.

Patienten mit geplanter Heim-parenteraler Therapie sollten vor Entlassung metabolisch stabil sein, physisch und emotional fähig sein, die Therapie durchzuführen, sowie ein adäquates häusliches Umfeld haben 36 . Nach der Entlassung sollten anfänglich engmaschige Kontrollen an einer spezialisierten Ambulanz erfolgen, um die klinischen und laborchemischen Parameter zu kontrollieren sowie die Therapieadhärenz und den Umgang mit dem Katheter und dem Equipment zu überprüfen.

Hinsichtlich oraler Flüssigkeitseinnahme werden isotone Getränke empfohlen, welche schluckweise getrunken sowie getrennt von den Mahlzeiten eingenommen werden sollten 37 . Die Zugabe von Zucker und Salz zur Flüssigkeit führt zur Aktivierung des Natrium-Glucose-Transporters im Dünndarm und nachfolgender passiver Wasserresorption und somit größerer Flüssigkeitsresorption wie wenn Wasser ohne Zusätze getrunken wird 38 . Die Einnahme von großen Mengen Flüssigkeit in kurzer Zeit führt zu vermehrten Verlusten über den Darm und sollte vermieden werden, auch wenn das Durstgefühl groß ist. Hypertone Lösungen (z.B. reine Fruchtsäfte, Trinknahrung) sollten ebenso wie hypotone Lösungen (z.B. reines Wasser) vermieden werden, da sie zu vermehrtem Durchfall führen können.

Es sollten mehrere kleine Mahlzeiten pro Tag eingenommen werden, eine Hyperphagie (unphysiologisch gesteigerte Nahrungsaufnahme) sollte angestrebt werden, um die Kalorienabsorption zu maximieren 22 (mehr Zufuhr = mehr Aufnahme). Bei erhaltenem Colon sollte eine fettreduzierte (ca. 20%) und kohlehydratreiche (ca. 60%) Ernährung angestrebt werden 39 , diese Empfehlungen sind jedoch im Alltag oft schwer umsetzbar. Bei endständigem Ileo-/Jejuno-Stoma ist keine besondere Diät empfohlen 40 . Trinknahrungen können die enterale Kalorienaufnahme signifikant steigern (siehe Praxistipp). Sollte das enthaltene Milchprotein zu vermehrten Blähungen führen, kann ein milcheiweißfreies Produkt probiert werden. Pankreasenzyme sollten auch zu den Trinknahrungen eingenommen werden.

## Komplikationen

Komplikationen tragen signifikant zur Mortalität und Morbidität der Kurzdarmpatienten bei und sollten daher so möglich vermieden, früh erkannt und effektiv behandelt werden.

### Katheterinfektionen

Eines der häufigsten Probleme bei Patienten mit chronischem Darmversagen und intravenöser Ernährung sind Katheterinfektionen 41 . Diese treten als Lokalinfektionen (Kathetereintrittsstelle), im subkutanen Verlauf (Tunnel, Port) oder als Katheter-assoziierte Sepsis auf. Auch in erfahrenen Zentren sind mit bis zu 1,09 Infektionsereignissen pro Katheter und Jahr zu rechnen 42 . Symptome einer Infektion sind lokale Rötung, Schwellung oder Sekretion, Fieber oder Schüttelfrost bei Verwendung des Katheters sowie laborchemisch erhöhte Entzündungsparameter nach Ausschluss anderer Infektionsursachen. Die Diagnostik sollte Blutkulturen aus dem Katheter sowie von peripher beinhalten, um eine Bakteriämie zu diagnostizieren. Im Ausnahmefall (simple Infektion mit Staphylokokkus aureus, koagulase-negativen Staphylokokken oder gram-negativen Bakterien oder schwierige Zugangssituation) kann eine Erhaltung des Katheters angestrebt werden und eine Therapie mit systemischen Antibiotika sowie lokaler Antibiose mittels Plombierung des Katheters durchgeführt werden 41 43 . Bei anhaltenden Infektionszeichen sowie in allen anderen Fällen ist eine Antibiose mit Entfernung des Katheters notwendig. Die Katheterspitze sollte anschließend mikrobiologisch untersucht werden. Die Anlage eines neuen Katheters sollte erst nach Abklingen des Infekts erfolgen. Die Antibiose sollte initial aufgrund des zu erwartenden Erregerprofils gewählt und nach Einlangen des mikrobiologischen Befundes angepasst werden. Häufig sind Infektionen mit Bakterien der Haut- sowie der Darmflora. Bei Infektionen mit Staphylokokken sowie Streptokokken sollte eine Endokarditis ausgeschlossen werden.

Präventive Maßnahmen zur Vermeidung von Katheterinfektionen sind regelmäßige Schulungen, Verwendung von Pflastern mit lokal antibakteriellen Substanzen (z.B. Chlorhexidin-Pflaster), Verwendung von nadelfreien Konnektoren und von Kappen mit Desinfektionsmitteln, Vermeidung von Blutabnahmen über den Katheter und Plombieren des Katheters mit Taurolidin 35 sowie ein pulsatives Spülen 44 .

### Andere Katheterkomplikationen

Ein weiteres Problem des Zugangs sind Katheter-assoziierte Thrombosen. Zur Verhinderung von Thrombosen werden Ultraschall-gezielte atraumatische Gefäßpunktionen sowie aseptische Techniken, weiches Kathetermaterial (Silikon), Katheterdurchmesser von maximal 1/3 des Gefäßlumens und Platzierung der Katheterspitze am Übergang Vena cava superior/rechter Vorhof empfohlen. Bei der Implantation soll auf korrekte Fixierung des Katheters geachtet werden. Ein PICC birgt aufgrund der Länge des Katheters ein erhöhtes Thromboserisiko und sollte nur bei spezieller Indikation gewählt werden. Thrombotische Okklusionen können mittels forcierter Spülung mit möglichst kleinen Spritzen behandelt werden, sowie mittels lokaler Lyse und endoluminalen Bürsten. Zur Prophylaxe wird forciertes, pulsatives Spülen nach jeder Verwendung mittels physiologischer Kochsalzlösung empfohlen 45 .

### Intestinal-failure associated liver disease/Darmversagen-assoziierte Lebererkrankung

Bei ungefähr 15–40% aller Patienten mit Langzeit-parenteraler Ernährung und chronischem Darmversagen kommt es zu dieser Lebererkrankung 46 . Die Ursachen sind multifaktoriell und beinhalten Nebenwirkungen der parenteralen Ernährung sowie negative Effekte des durch die Erkrankung veränderten Stoffwechsels per se. Das klinische Bild ist meist gemischt steatotisch und cholestatisch und kann progredient bis hin zu einer Leberzirrhose sein. Falls laborchemisch erhöhte Leberparameter auffällig sind, sollen andere Ursachen für eine Hepatopathie ausgeschlossen werden 23 . Zur Vermeidung sollten die orale Ernährung gefördert und auf Soja-öl basierte Fette auf <1g/kg/d reduziert werden 35 . Weiter sollte die parenterale Ernährung auf das mögliche Minimum reduziert und über wenige Stunden pro Tag gegeben werden, auch Tage ohne parenterale Ernährung sind hilfreich 23 47 . Eine intestinale Rekonstruktion trägt zu Verbesserung der Leberfunktion bei, falls dabei eine signifikante Verbesserung der Darmlänge erreicht werden kann 23 .

### Gallensteine/Cholelithiasis

Patienten mit KDS entwickeln häufig Gallensteine. Eine enterale Ernährung trägt zur Prävention bei 48 . Bei Auftreten von Komplikationen erfolgt die Behandlung gleich wie bei Darm-Gesunden.

### Small intestinal bacterial overgrowth (SIBO)

Durch die gestörte Darmmotilität bei Adhäsionen, Stenosen, Dilatationen und blinden Darmschlingen kommt es häufig zu bakteriellem Überwuchs 49 . Bei typischer klinischer Symptomatik (in 1. Linie Gasbildung, aber auch Völlegefühl, vermehrte Durchfälle) sollte ein Therapieversuch mittels Antibiose durchgeführt werden (z.B. Rifaximin 1200mg–1600mg/d für 14 Tage) 50 51 . Dies kann auch zu einer verbesserten Darmmotilität und verbesserter Vitamin B12 und Nährstoffaufnahme Aufnahme führen.

### Niereninsuffizienz, Nierensteine

Ein Mangel an Flüssigkeit durch die hohen enteralen Verluste kann zu einem akuten Nierenversagen führen. Hingegen ist die Entstehung einer chronischen Niereninsuffizienz multifaktoriell, chronische oder wiederholte Dehydrierung, wiederholte Katheter-assoziierte Sepsis Episoden sowie nephrotoxische Medikamente und das fortschreitende Alter tragen zu einer reduzierten Nierenfunktion bei 52 . Regelmäßige Kontrollen der Nierenwerte sowie der Harnmenge sind essenziell. Eine metabolische Azidose sollte ausgeglichen werden. Gerade bei erhaltenem Colon ist das Risiko für Oxalat-Nierensteine stark erhöht 53 . Bei rezidivierenden Nierensteinen sollte daher auf eine Fett- und Oxalat-reduzierte Ernährung geachtet werden und zusätzlich Calcium oder Magnesium zugeführt werden da dies an Oxalat bindet und somit die Aufnahme reduziert 13 . Die Therapie einer Niereninsuffizienz und von Nierensteinen sollte leitliniengerecht erfolgen.

### Osteoporose (Chronic intestinal failure associated metabolic bone disease)

Nicht nur Vitamin D-Mangel und niedrige Spiegel von Calcium und Phosphat, sondern auch verschiedene andere Faktoren wie chronische Entzündung, Infektionen und Medikamente führen häufig zu einer Osteopenie oder Osteoporose. Es sollte ein jährliches Screening mittels Knochendichtemessung erfolgen 23 . Vor Einleitung einer spezifischen Therapie sollten die Vitamine und Spurenelemente optimiert werden, falls nicht anders möglich, auch parenteral.

### Psychologische Folgen

Die Erkrankung per se sowie die dadurch notwendigen Arztbesuche und Therapien beeinflussen das Leben der Patienten stark. Es kann zu verminderter Lebensqualität, Isolation, chronischer Müdigkeit, Depressionen und Belastungsstörungen kommen. Ebenso weitreichend sind die Folgen für das Berufs- und Privatleben. Eine frühzeitige Vorstellung beim Facharzt für Psychiatrie, eine Anbindung an klinische Psychologen sowie psychosoziale Unterstützung (z.B. auch durch Selbsthilfegruppen) ist wichtig.

## Zusammenfassung

Kurz zusammengefasst ist das Kurzdarmsyndrom ein seltenes und komplexes Krankheitsbild, das aufgrund der Symptome und der notwendigen Therapien die Lebensqualität und Lebenserwartung erheblich einschränken kann. Die multidisziplinäre Mitbetreuung an einem spezialisierten Zentrum ist von großer Notwendigkeit, um den Krankheitsverlauf positiv zu beeinflussen 54 . Jedoch auch ein gutes Netzwerk an niedergelassenen Ärzten ist essenziell, um eine engmaschige Betreuung zu ermöglichen. Eine Unabhängigkeit von parenteraler Unterstützung durch optimale medikamentöse und diätologische Einstellung führt zu weniger Komplikationen und verbesserter Lebensqualität. Die Anbindung an eine Selbsthilfegruppe ( www.chronisch.at ) sollte allen Patienten empfohlen werden.

## Notes

Praxistipp Durchfall kann auch durch andere Ursachen außer der Malabsorption im Rahmen des KDS (mit-)verursacht werden. Bei Veränderungen der Menge/Frequenz/Konsistenz sollte dies bedacht und entsprechend leitlinienkonform abgeklärt werden (infektiös, Laktoseintoleranz, SIBO, Zöliakie etc.).

## Notes

Praxistipp Nur ca. 1/3 bis 1/4 der üblichen Mannitolmenge sollte für CT/MR Enterografie verwendet werden sowie ein kürzerer Abstand zwischen Verabreichung und Durchführung der Bildgebung

## Notes

Praxistipp Einnahme von Loperamid ca. 30 Minuten vor einer Mahlzeit optimalerweise in einer Galenik, welche rasch aufgenommen wird (z.B. Schmelztabletten)

## Notes

Praxistipp Da auch die Aufnahme von Medikamenten gestört sein kann, sollte jede Art von Medikament möglichst in nicht-retardierter Formulierung (so verfügbar) und in Tropfenform oder mittels Schmelztabletten gegeben werden, um eine vollständige Aufnahme zu garantieren. So verfügbar, sollten Medikamentenspiegel gemessen werden.

## Notes

Praxistipp Medikamente regelmäßig auf Wirksamkeit prüfen und nicht wirksame absetzen!

## Notes

Praxistipp Eine gute Darmvorbereitung ist für eine Koloskopie essenziell. Ob und wie bei Kurzdarmpatienten die Vorbereitungslösung reduziert werden kann, ist unbekannt. Im Zweifel sollte die normalerweise empfohlene Menge der Darmspüllösung eingenommen werden, um eine sichere und sinnvolle Koloskopie zu ermöglichen.

## Notes

Praxistipp
Vitamine und Spurenelemente müssen oft in weitaus höherer Dosis als üblich zugeführt werden, um normale Werte zu erreichen. Siehe Tab. 3 .

## Notes

Praxistipp Bei anhaltender Hyperglykämie trotz Optimierung der Medikamente und Ernährung kann dem Ernährungsbeutel kurzwirksames Insulin zugefügt werden. Beginnend mit: diabetische Stoffwechsellage 1 IE pro 10g Glucose, normale Stoffwechsellage 1 IE pro 20g Glucose. Weitere Titration je nach Bedarf. Eine kontinuierliche Blutzuckermessung mittels Sensor wird ausdrücklich empfohlen.

## Notes

Praxistipp Das pulsative Spülen (rasch und fest mit jeweils 1–2ml für insgesamt 3×10ml) des Katheters mit Kochsalzlösung nach jeder Verwendung kann die Rate an Katheterinfektionen reduzieren. Die Klemme ist zu schließen, bevor die Kochsalzspritze vollständig entleert wurde, damit nicht nach dem Rückflussprinzip wieder Blut in den Katheter gezogen wird.

## Notes

Praxistipp
Optimalerweise haben Patienten immer eine Trinkflasche bei sich und trinken alle 10–15 Minuten wenige Schlucke. Eine speziell entwickelte Trinkflasche zur erleichterten Herstellung von isotonen Getränken ist unter www.chronisch.at gegen Versandkostenerstattung erhältlich (Stand Dezember 2024).

## Notes

Praxistipp
Rezepte für isotone Getränke sind auf der Homepage der Selbsthilfegruppe „Chronische Experten“ zu finden ( www.chronisch.at )

## Notes

Praxistipp Trinknahrungen schluckweise immer mit einem zusätzlichen Schluck Wasser (oder 1:1 mit Wasser verdünnt) einnehmen, um zusätzliche Durchfälle zu vermeiden und die Aufnahme zu maximieren

## Notes

Praxistipp Zur Vermeidung von Nierensteinen kann Magnesiumcitrat den Getränken hinzufügt und über den ganzen Tag verteilt konsumiert werden
